# Antimicrobial resistance in breeding pigs in Germany – differences between 2015 and 2023

**DOI:** 10.1186/s40813-026-00508-2

**Published:** 2026-04-28

**Authors:** Bernd-Alois Tenhagen, Tobias Lienen, Istvan Szabo, Carolina Plaza-Rodriguez, Mirjam Grobbel

**Affiliations:** Department Biological Safety, German Federal Institute for Risk Assessment, Max Dohrn Str. 8-10, 10589 Berlin, Germany

## Abstract

**Background:**

Direct contact with their offspring during the first few weeks makes sows a potential source of resistant bacteria for fattening pigs and food products derived from pigs. Moreover, staff working with breeding pigs are exposed to their bacterial flora which also includes zoonotic pathogens and antimicrobial resistant bacteria. In this study we investigated the antimicrobial resistance of *E. coli* and the prevalence and the antimicrobial resistance of *Salmonella enterica*, cephalosporin-resistant *Escherichia coli* (CTX^R^-*E. coli*) and methicillin-resistant *Staphylococcus aureus* (MRSA) in breeding pigs in Germany in 2015 and 2023 to identify potential changes in the prevalence and resistance over time during a period of reduction of antimicrobial use in farm animals in Germany.

**Results:**

Resistance in *E. coli* to tetracycline and sulfamethoxazole and the prevalence of CTX^R^-*E. coli* decreased in breeding herds between 2015 and 2023 in Germany. The proportion of *E. coli* that were susceptible to all tested substances increased. In contrast, there were no changes in the herd prevalence of *Salmonella* and MRSA. Resistance of *E. coli* to some antimicrobials was associated with the presence of MRSA in the herd and to herd size. Larger herds were more prone to harbour MRSA and CTX^R^-*E. coli.*

**Conclusions:**

Results demonstrate a minor decrease in AMR in *E. coli* from breeding pigs in Germany but no decrease in the prevalence of *Salmonella* or MRSA.

## Background

Antimicrobial resistance (AMR) in food producing animals is an important issue for both human and veterinary public health. Foodborne transmission of resistant bacteria is evident in zoonotic pathogens like *Salmonella* (*S.*) *enterica* [1] or thermophilic *Campylobacter* spp [2]. However, it can also be assumed to occur in non-pathogenic bacteria such as *Escherichia* (*E.*). *coli*, a commensal bacterium of the mammalian gut flora. Nonetheless, the role of these *E. coli* in contributing to AMR in *E. coli* from humans is difficult to establish [3, 4].

Breeding pigs are not a major source of direct exposure for humans, but occupationally exposed individuals (such as farms workers housing such pigs and abattoir staff handling live animals) are at higher risk of colonization [5–7]. The major role of breeding pigs is not food production but producing offspring that will then, after the end of its productive life, enter the food chain. Still, breeding pigs have to be considered as a relevant source of bacteria colonizing their offspring after birth as they live in close contact for the first couple of weeks [8, 9]. Hence, the bacterial flora of breeding pigs may be an important primary source of resistant bacteria for fattening pigs and therefore for food products derived from pigs. A sow will influence the bacterial flora of an average of 13 piglets per litter and of four to five litters in its lifespan and therefore has an important multiplier effect [10]. Moreover, sows remain in the herd for long and may therefore be an important reservoir of resistant bacteria [8, 9]. For this reason, it is relevant to also investigate and potentially control the prevalence and trend of resistant bacteria in breeding pigs to limit the role of sows as a source of resistant bacteria for their offspring.

Resistance in bacterial isolates from breeding pigs has often been described as lower as compared to fattening pigs [11]. However, some exceptions have been noted with (fluoro)quinolone-resistant *E. coli*, which are more frequent on breeding farms. Environmental samples such as boot swabs or dust from breeding farms may also reflect AMR in piglets [12]. The treatment frequency of breeding pigs in Germany with antimicrobials is lower than that of suckling piglets, weaners or fatteners [13–15]. However, during lactation they may be co-exposed when their offspring is treated.

From a public health perspective, the occurrence of *S. enterica* is important not only on account of its resistance to antimicrobials but also as a foodborne pathogen, as it is still one of the most common causative agents of foodborne zoonotic infections in humans in Europe [16]. The pig meat chain has been pointed out as an important source, especially with respect to *S.* Typhimurium [17]. *Salmonella* prevalence in sows is frequently studied, although models show that interventions at the sow level are likely of limited value when it comes to reducing the presence of *Salmonella* in pork meat at retail [18]. Still, in addition to their role as a source of resistant bacteria, sows are an important reservoir for the colonization of piglets with *Salmonella* as they may carry *Salmonella* for a long time [19].

*E. coli* is considered a suitable indicator of AMR in animal populations [20] and therefore a relevant bacterial species to be investigated.

In recent years, the occurrence of specific multiresistant strains of bacteria, such as *E. coli* resistant to 3rd generation cephalosporins (CTX^R^-*E. coli*) or methicillin-resistant *Staphylococcus aureus* (MRSA), has been put in the focus. These bacteria are involved in substantial resistance problems in healthcare settings and should be investigated to identify the role of animal derived strains in medical settings early. However, to date animal derived strains seem to play a minor role in those settings [21].

Livestock associated MRSA have been found in pigs in Germany for more than 20 years [22]. Along with these findings, MRSA have been also frequently detected in pig farmers in Germany [23]. In a European study conducted in 2008, Germany was among the countries with the highest prevalence of MRSA in sow herds [24, 25]. Therefore, its prevalence has been investigated in the framework of the National Zoonoses Monitoring program twice in the last 10 years, i.e., in 2015 and in 2023. It has been pointed out that control of MRSA in pig farms is complex especially due to the high risk of re-stocking farms with positive animals [26]. Therefore, any attempt to control MRSA needs to start with eliminating the bacteria from the breeding herds. Still, the likelihood of successfully clearing MRSA from the breeding herds is very low in countries with a high herd prevalence of MRSA.

Third and 4th generation cephalosporins and (fluoro)quinolones are considered highest priority critically important antimicrobials by the World Health Organization [27] and have been categorized under Category B by the European Medicines Agency [28]. Therefore, their use should be avoided in farm animals. In Germany, legal action has been taken in 2018 to specifically reduce the use of these drugs in farm animals [29]. While the use of these cephalosporins has decreased in animals in Germany, no effect on the prevalence of extended-spectrum beta-lactamase- or ampicillinase class C beta-lactamase-producing *E. coli* (ESBL/AmpC-producing *E. coli*) in fattening pigs at slaughter could be found until 2021 [30]. However, cephalosporins are more likely used in breeding animals than in fattening pigs as there is no oral formula of these substances on the market in Germany [14, 15].

In this study we investigated the AMR of *E. coli* and the prevalence and AMR of *S. enterica*, cephalosporin-resistant *E. coli* (CTX^R^-*E. coli*) and MRSA in breeding pigs in Germany in 2015 and 2023 to identify potential changes in the prevalence and resistance over time during a period of reduction of AMU in farm animals in Germany. Our hypothesis was that resistance in the isolates and the prevalence of the specific resistant bacteria might decrease as antimicrobial sales to veterinarians in Germany decreased substantially from 805 tonnes to 529 tonnes between 2015 and 2023 [31].

## Methods

Boot sock samples and composite faecal samples (one sample of each per herd) were collected in randomly selected breeding pig herds in Germany in the framework of the National Monitoring of zoonotic pathogens and resistant microorganisms in the food chain. Sow herds had a minimum of 50 breeding pigs. Sampling was distributed across the federal states proportionate to their breeding pig population. Samples were collected in the areas where pregnant sows are housed. This was done to minimize the effect of other age groups on the farms (i.e. suckling piglets or weaned piglets) on the results of the investigation. Sampling frames in 2015 and 2023 were independent from each other, i.e., there was no direct connection between the farms in the two years. Samples were collected by the local veterinary authorities or under their supervision.

### Laboratory procedures

Samples were shipped to the regional state laboratories. In the regional laboratories, boot sock samples were suspended in Müller-Hinton Broth with 6% NaCl (for MRSA) and faecal samples in buffered peptone water (BPW) and investigated according to harmonized protocols.

*Salmonella* isolation was performed following the procedure for samples from the primary production stage given in ISO 6579-1:2017. The isolation of *E. coli* was done using both, non-selective and selective methods. For the non-selective isolation of *E. coli* no specific method was given. The selective isolation of CTX^R^ *E. coli* was performed following protocols given by the European Reference Laboratory for Antimicrobial Resistance (EURL-AR) where a non-selective enrichment in buffered peptone water is followed by a selective culture on MacConkey agar supplemented with 1 mg/L cefotaxime [32]. Over time, the details of the protocols changed slightly. However, no significant change in sensitivity was expected by these changes.

Samples were tested for MRSA using a two step enrichment protocol (2015) and a one step enrichment protocol (2023), based on the respective recommendations of the EURL at the time. Briefly, boot socks were incubated in 100 ml of Müller-Hinton broth containing 6% NaCl (37 °C, 24 h). In 2015, one ml was transferred to 9 ml of a second enrichment broth containing 3.5 mg/l cefoxitin and 50 mg/l of aztreonam. This broth was subsequently incubated for another 24 h at 37 °C. This second enrichment was omitted in 2023. Finally, the broth was streaked on commercial selective media for MRSA. Suspect colonies were sent to the NRL for confirmation and further characterization. No major differences in sensitivity were expected for the two methods.

Isolates were submitted for confirmation, further characterization and antimicrobial susceptibility testing to the respective National Reference Laboratories (NRLs) at the German Federal Institute for Risk Assessment (BfR) in Berlin. Staphylococci were sent to the NRL for coagulase-positive staphylococci incl. *S. aureus*, *Salmonella* isolates were sent to the NRL for *Salmonella* and *E. coli* were sent to the NRL for Antimicrobial Resistance.

At the NRL, the species was confirmed using MALDI TOF. The respective resistance characteristics of the isolates that were isolated using selective methods were also confirmed. Isolates of *Salmonella* and *E. coli* were tested for their susceptibility to antimicrobials as defined in Commission Implementing Decision (CID) 2013/652 (EU) (isolates from 2015) and (EU) 2020/1729 (isolates from 2023), respectively. The different decisions did not differ in technical instructions. However, the test ranges were slightly adjusted and amikacin was included in the panel of CID (EU) 2020/1729. There was no resistance to amikacin and therefore it was not included in the analyses. The prescriptions of the CID are in line with ISO 20776-1:2019 (in 2023). Minimum inhibitory concentrations (MIC) were uniformly interpreted according the epidemiological cut off values as provided in CID (EU) 2020/1729. They are displayed in Table 2.

Resistance of staphylococci was tested according to technical specifications published by the European Food Safety Authority (EFSA) in 2012 [33]. Here, MIC were evaluated according the most recently published ECOFFs by EUCAST (Table 3). Isolates with values above the ECOFFs are further defined as resistant, isolates with resistance to more than two different classes of antimicrobials were defined as Multi Drug Resistant (MDR) [34].

Antimicrobial susceptibility testing was performed by broth microdilution according to international guidelines (ISO 20776-1:2006 / CLSI M07 Ed 12). It was carried out using a commercial standardized antibiotic panel (Sensititre EUST scheme from Thermo Fisher Scientific, UK) that is recommended by the European Food Safety Authority (EFSA) for resistance monitoring in MRSA from livestock and food. For quality control of resistance testing, the *S*. *aureus* strain ATCC 29,213 and *Enterococcus faecalis* strain ATCC 29,212 were used. In 2023, CTX-R isolates of *E. coli* from the selective media were not tested for resistance to other substances phenotypically but underwent whole genome sequencing.

All MRSA isolates were typed according to the staphylococcal protein A (*spa*) gene sequence [35]. Sequencing of purified *spa* gene products was performed by Eurofins laboratories (Eurofins Genomics, 85560, Ebersberg, Germany). The sp.a.-types were assigned using Ridom SeqSphere+ version 10.5.2. Analysis of the sequence type (ST) by multilocus sequence typing was done according to [36].

For whole genome sequencing (WGS) of MRSA, DNA of 1 ml culture was extracted using the Qiagen DNeasy Blood and Tissue Kit (Qiagen, Germany) according to the manufacturer´s protocol modified by adding 10 µl lysostaphin (Sigma Aldrich, USA) to the lysis buffer. For CTX^R^ *E. coli* of 2023, DNA was extracted using the PureLink Genomic DNA Mini Kit (Invitrogen, USA) following the manufactures instructions.

For both, CTX^R^ *E. coli* and MRSA the DNA library was prepared using an Illumina DNA Prep kit (Illumina Inc., USA) and the 150 bp paired-end sequencing run was performed on an Illumina NextSeq 500 instrument. Raw Illumina reads were trimmed and de novo assembled with the in-house developed AQUAMIS pipeline [37]. Assignment of sequence types was done with the in-house developed Bakcharak pipeline (https://gitlab.com/bfr_bioinformatics/bakcharak).

### Data analysis

Only data from farms with available test results for all four parameters (*E. coli*, CTX^R^ *E. coli*, MRSA, and *Salmonella*) were included. Unfortunately, not all positive samples yielded isolates, either because they were not submitted to the NRLs at BfR or could not be re-grown upon arrival. Those samples were considered positive, but the isolates were not included in the analysis of AMR or sequencing results. On the other hand, isolates that were submitted to the NRLs, but were not accompanied by a respective complete data set were also excluded from the analysis. Finally, samples from 679 farms were included in the analysis, 346 from 2015 to 333 from 2023.

The AMR of the isolates to individual antimicrobials, their multi drug resistance and the prevalence of the specific resistant bacteria were analysed in relation to the prevalence of *Salmonella*, MRSA and CTX^R^-*E. coli* in the herd, the sampling year, the region and herd size using binary logistic regressions with resistance, MDR or presence of the bacterial group as the binary outcome (0 susceptible/absent, 1 resistant, present). For resistance of *E. coli*, the presence or absence of MRSA, CTX^R^-*E. coli* and *Salmonella* was included in the model. For isolates of *Salmonella*, MRSA and CTX^R^-*E. coli* the presence of the respective bacteria dropped out of the models, as all Salmonella were from *Salmonella* positive herds etc.

For the majority of the farms, information on herd size was available. Herd size was categorized as small (< 150 sows), medium (150–1000 sows) and large (> 1000 sows). This was done to avoid a substantial influence of the very large herds on the outcome of the regression analyses. To be able to include data from herds with unknown herd size in the regression models, those were categorized as “unknown” and formed a separate category for the analysis. Region was categorized into Northwest, Southwest and East as described previously [38].

The number of antimicrobial classes that the MRSA-isolates were resistant to was compared between the years using Mann-Whitney U Test. For *E. coli* and *Salmonella*, the proportion of isolates that were resistant to at least one of the substances and to more than two substance classes (MDR) was compared using the same logistic regression model as for the individual substances.

All analyses were carried out using IBM SPSS Statistics Version 26. α was set at 0.05.

## Results

Overall, 377 farms were visited in 2015 and 354 in 2023. After exclusion of farms with missing data 346 and 333 farms remained in the analysis. The prevalence of *Salmonella enterica*, CTX^R^ *E. coli* and MRSA is displayed in Table 1.

### *Salmonella* spp.

*Salmonella* was infrequently detected in the samples (4.6%). Prevalence did not differ significantly between 2015 (4.9%) and 2023 (4.2%, *p* > 0.05, OR 0.963, *p* = 0.431) nor between herd sizes or regions. In both years, *S.* Derby was the most frequent serovar with more than half of the isolates assigned to this serovar (9/17 and 7/13, respectively). *S.* Typhimurium was the second most frequent in 2015 but was not detected in 2023. In 2015, there were two isolates of the monophasic variant and only one biphasic *S.* Typhimurium. The other serovars differed between years, i.e., all serovars but *S.* Derby identified in 2015 were not found in 2023, and vice versa. There were two isolates of *S*. Infantis in 2015. All other serovars were only identified once: *S.* Goldcoast and *S.* Mbandaka in 2015, *S.* Give, *S*. London, *S*. Rissen, *S*. Senftenberg and *S*. Utah in 2023. One isolate per year was serologically rough.

Across all serovars, there was a significant increase in resistance to the (fluoro)quinolones with 0/13 resistant isolates in 2015 and 4/17 in 2023 (*p* = 0.026, Fishers exact test, Table 2). Otherwise, no significant difference was observed. Resistance to the (fluoro)quinolones was only observed in *S.* Derby, *S*. Rissen and *S*. London. Besides *S*. Derby, resistance to sulfamethoxazole was observed in all *S*. Typhimurium isolates in 2015 and in the isolate of *S*. Rissen and *S*. Senftenberg in 2023.

Resistance of *S.* Derby to antimicrobials did not differ significantly between years (data not shown). There was no resistance to most of the antimicrobials. Resistance was observed to sulfamethoxazole in both years, to ampicillin and tetracycline in 2015 and to ciprofloxacin and nalidixic acid in 2023.

**Table 1:** Proportion of positive samples for *Salmonella* spp., Cefotaxim-resistant *E. coli* (CTX^R^ *E. coli*) and Methicillin-resistant *Staphylococcus aureus* (MRSA)

Herd size	No of samples			CTX^R^ *E. col*i^1^			MRSA		* Salmonella enterica *	
	2015	2023	Total	2015	2023		2015	2023	2015	2023
< 150	99	78	177	47.5	33.3		19.2	16.7	3.0	1.3
150–1000	207	173	380	55.6	48.6		29.0	23.7	4.8	4.6
> 1000	30	50	80	63.3	64.0		23.3	48.0	10.0	10.0
unknown	10	32	42	40.0	37.5		30.0	21.9	10.0	0.0
Total	346	333	679	53.5	46.2		25.7	25.5	4.9	4.2

^1^ Prevalence of cefotaxime resistant *E. coli* using selective isolation according to [32]

### Methicillin-resistant *S. aureus*

MRSA were observed in breeding pigs in both years with a similar prevalence (25.7% vs. 25.5%, *p* = 0.718). Detection of MRSA in the herd was positively associated with the detection of CTX^R^ *E. coli* in the herd (OR 1.584, *p* = 0.012). Positive findings were less frequent in the Southwest than in the Northwest (OR 0.574, *p* = 0.032). Large herds (> 1000 sows) were more likely MRSA-positive than small herds (< 150 sows, OR 3.189, *p* = 0.002).

In both years, most isolates were assigned to the clonal complex CC398 while a small proportion was assigned to other complexes. In 2015, three isolates were assigned to ST9 (*spa*-type t1430). In 2023, *spa*-type t1430 was not observed but six of the seven non-CC398 isolates were *spa*-type t899 and by WGS assigned to ST9. One other isolate was *spa*-type t10204 and assigned to CC1.

Overall, antimicrobial resistance of the MRSA-isolates underwent some significant changes between 2015 and 2023 (Table 3). The frequency of resistance to chloramphenicol decreased significantly, while the proportion of MRSA-isolates that were resistant to trimethoprim, clindamycin, quinupristin/dalfopristin and tiamulin increased. The number of antimicrobial classes that the isolates were resistant to tended to be higher in 2023. However, the difference between the years was not significant (*p* = 0.068). Only resistance to ciprofloxacin differed between herd sizes and regions (Table 3). For all other resistance traits that could be included in the logistic regression model, no significant associations other than with year were observed.

### Indicator *E. coli*

Resistance of the 404 *E. coli* isolates to 14 antimicrobials is displayed in Table 2. It was most frequent to tetracycline, sulfamethoxazole, ampicillin and to trimethoprim. Resistance to each of the other antimicrobials was found in less than 10% of the isolates.

Around 50% of the isolates were susceptible to all 14 substances. This proportion was lower in 2015 than it was in 2023 (OR: 0.94, *p* = 0.029). In contrast, the proportion of multidrug-resistant (MDR) isolates did not differ between the years. Resistance to tetracycline (OR 0.938, *p* = 0.041) and sulfamethoxazole (OR 0.932, *p* = 0.042) was less frequent in 2023 than in 2015. Herd size was also associated with resistance of *E. coli* to individual antimicrobials. In large herds (> 1000 sows), isolates were more often resistant to tetracycline (OR 2.682, *p* = 0.035), trimethoprim (OR 4.146, *p* = 0.009) and sulfamethoxazole (3.091, *p* = 0.25) as compared to isolates from small herds. More of the isolates were MDR in large herds than in small herds (OR 3.294, *p* = 0.026). Finally, the detection of MRSA in the herd was also associated with higher resistance rates in the *E. coli* isolates. This was found for ampicillin (OR 1,926, *p* = 0,011), trimethoprim (OR 2.119, *p* = 0.007), tetracycline (OR 2.146, *p* = 0.002) resistance to at least one antimicrobial (OR 2.520, *p* = 0.036) and for MDR (*p* = 1,936, *p* = 0,018).

### CTX^R^*E. coli*

Although resistance to the 3rd generation cephalosporins was infrequent in the indicator *E. coli* that were harvested from non-selective media and did not change significantly over time, a substantial but decreasing proportion of the samples (2015: 53.9%, 2023: 46.3%, *p* < 0.05, OR 0.74 (0.55–0.99)) contained cephalosporin-resistant *E. coli* when examined by selective isolation. Their detection was associated with the detection of MRSA (see above). It was more frequent in medium sized and large herds than in small herds (OR = 1.50, *p* = 0.037 and OR 2.283, *p* = 0.015, respectively).

In 2015, isolates from the selective isolation of CTX^R^ *E. coli* were more frequently phenotypically resistant to other antimicrobials than randomly tested *E. coli* (Table 3). Only for three substances (meropenem, colistin and tigecycline), the proportion of resistant isolates did not differ. To all of the three substances, a maximum of one isolate was resistant. The resistance of the individual isolates of the CTX^R^ *E. coli* could mostly not be associated to individual risk factors. However, resistance to ciprofloxacin in these isolates was more frequent if MRSA had also been detected in the herd (OR 2.213, *p* = 0.029).

**Table 2:** Percentage antimicrobial resistance in *Salmonella enterica*, *E. coli* and selectively isolated 3rd generation cephalosporin resistant *E. coli* (CTX^R^ *E. coli*) from breeding pigs in Germany in 2015 and 2023

Species	Salmonella enterica			*E. coli*		CTX^R^ *E. coli* ^*1*^	*E. coli*
**Year**	2015	2023	ECOFF	2015	2023	2015	ECOFF^2^
**No of isolates**	17	13	mg/l	242	162	164	mg/l
Ampicillin	29.4	7.7	8	25.2	22.2	100.0^***^	8
Azithromycin	0.0	0.0	16	2.5	1.2	15.9^***^	16
Chloramphenicol	11.8	7.7	16	5.0	8.0	16.5^**^	16
Ciprofloxacin	0.0	30.8^a^	0.06	6.6	6.2	42.1^***^	0.06
Colistin	0.0	0.0	2	0.4	0.0	0.0	2
Cefotaxime	0.0	0.0	0.5	1.2	1.2	100.0^***^	0.25
Gentamicin	0.0	0.0	2	3.3	1.2	14.0^***^	2
Meropenem	0.0	0.0	0.125	0.0	0.0	0.0	0.125
Nalidixic acid	0.0	30.8^a^	8	5.0	4.3	39.6^***^	8
Sulfamethoxazole	35.3	23.1	256	25.6	18.5^a^	62.8^***^	64
Ceftazidime	0.0	0.0	2	0.8	1.2	97.0^***^	0.5
Tetracycline	29.4	7.7	8	36.8	25.9^a^	47.6^*^	8
Tigecycline	0.0	0.0	0.5	0.4	0.0	0.0	0.5
Trimethoprim	23.5	7.7	2	19.0	19.1	59.8^***^	2
MDR (> 2 classes)	35.3	7.7		19.4	17.3	84.8^***^	
Resistant ≥ 1 class	35.3	46.2		54.5	42.6^a^	100.0^nt^	

^1^ isolated using selective media

^2^ epidemiological cut off value

^a^ significantly different between 2015 and 2023

^*^ significantly different between *E. coli* and CTX^R^-*E. coli* in 2015 *(p < 0.05)*

^**^ significantly different between *E. coli* and CTX^R^-*E. coli* in 2015 *(p < 0.01)*

^***^ significantly different between *E. coli* and CTX^R^-*E. coli* in 2015 *(p < 0.001)*

^Nt^ not tested

In 2023, the most frequent resistance gene associated with resistance to 3rd generation cephalosporins from sows was *bla*_CTX−M−1_ (87 isolates), followed by *bla*_CTX−M−15_ (17 isolates), *bla*_SHV−12_ (8 isolates) and *bla*_CTX−M−14_ (5 isolates). The gene *bla*_TEM−1_ was detected in 44 isolates, 26 of which also carried the gene *bla*_CTX−M−1_. Other TEM-1 were found in combination with *bla*_CTX−M−15_ [6], *bla*_CTX−M−14_ [3] and *bla*_SHV−12_ [3]. AmpC producing genes were rare, with only one *bla*_CMY−2_ and one *bla*_DHA−1_. AmpC point mutations were more frequent with C-42T being most frequent [14] and INS-13 [1] and T-32 A [3] occurring only sporadically.

**Table 3:** Percentage resistance in methicillin-resistant *Staphylococcus aureus* (MRSA) from breeding pigs in Germany to other antimicrobials

Antimicrobial^1^	2015 (*N* = 82)	2023 (*N* = 76)	Total	ECOFF^2^ mg/l	Odds ratio (year)	95% confidence interval of odds ratio (year)	*p*-value (year)
**Chloramphenicol**	**13.4**	**3.9**	**8.9**	**16**	**0.757**	**0.618–0.928**	**0.007**
Ciprofloxacin^3^	30.5	28.9	29.7	1	0.999	0.906–1.101	0.979
Gentamicin	13.4	5.3	9.5	2	0.858	0.728–1.010	0.066
Sulfamethoxazole	11.0	1.3	6.3	128	0.774	0.593–1.011	0.060
Tetracyclin	93.9	94.7	94.3	1	0.979	0.813–1.178	0.820
**Trimethoprim**	**57.3**	**73.7**	**65.2**	**2**	**1.102**	**1.004–1.220**	**0.040**
**Clindamycin**	**59.8**	**80.3**	**69.6**	**0.25**	**1.133**	**1.027–1.249**	**0.012**
Erythromycin	48.8	57.9	53.2	1	1.044	0.957–1.139	0.328
Kanamycin	15.9	6.6	11.4	8	0.880	0.759–1.020	0.089
Rifampicin^4^	1.2	1.3	1.3	0.016	nt		nt
Fusidic acid^4^	8.5	0	4.3	0.5	nt		nt
Streptomycin	39.0	27.6	33.5	16	0.932	0.850–1.021	0.137
**Quinupristin/Dalfopristin**	**30.5**	**56.6**	**43.0**	**1**	**1.132**	**1.034–1.239**	**0.007**
**Tiamulin**	**37.8**	**59.2**	**48.1**	**2**	**1.113**	**1.020–1.215**	**0.016**
Vancomycin^4^	1.2	0.0	0.6	2	nt		nt

^1^All isolates were susceptible to linezolid (ECOFF 4 mg/l) and mupirocin (1 mg/l) and resistant to penicillin (ECOFF 0.125 mg/l) and cefoxitin (ECOFF 4mg/l)

^2^Epidemiological cut off value. Minimum inhibitory concentrations above the ECOFF were considered resistant

^3^Resistance to ciprofloxacin was significantly associated with presence of CTX^R^ *E. coli* in the herd (OR 3.466, p=0.003) and was lower in the Southwest than in the Northwest (OR 0.088, p=0.027)

^4^Models could not be calculated because of too few positive samples

## Discussion

### Changes in AMR of *E. coli*

AMR in *E. coli* isolates from breeding pigs in Germany dropped significantly for two of the 14 investigated substances, i.e. sulfamethoxazole and tetracycline. In line with that, the proportion of isolates that were susceptible to all tested substances increased and the proportion of positive samples for CTX^R^-*E. coli* decreased (see below). No significant increase in resistance proportion was observed in *E. coli* to any of the tested substances. A numeric but non-significant increase was only observed for three substances. One of them was chloramphenicol, a substance that has been banned from use in farm animals decades ago.

The decrease in resistance observed in breeding pigs is in line with a decrease in AMR in *E. coli* from fattening pigs at slaughter reported in the framework of the National antimicrobial minimization concept in Germany [39]. This minimization concept targets the quartile of farms with the highest antimicrobial use in the respective animal population. These farms have to present a management plan to the regional veterinary authority with the aim of reducing antimicrobial use. Until 2022, the minimization concept included weaned piglets up to 30 kg and fattening pigs above 30 kg of body weight. For them, treatment frequency, the measure used in Germany for the benchmarking system, dropped significantly between 2014 and 2023 [15].

Overall antimicrobial sales to veterinarians in Germany have also decreased substantially between 2015 and 2023 [40]. Sows were, however, not considered when the minimization concept was first designed. This was based on the assumption, that the meat production animals were more prone to high AMU. Therefore, measures taken in those populations were more likely to produce significant gains. Sows were at that time considered a lower priority being further away from meat harvesting. They also had a lower consumption of antimicrobials in research projects than weaners or fattening pigs [13, 41–43]. In line with this assumption, resistance in *E. coli* was much lower in *E. coli* from sows than from weaner pigs in 2015, with 45.2% completely susceptible isolates in sows vs. only 29.6% in weaners [44]. This was in line with data from other countries e.g. Switzerland with 22.7% completely susceptible isolates in sows and 17.7% in weaners in 2004/05 [45].

The reduction in resistance of *E. coli* from sows in Germany is also in line with the reduced treatment frequencies in sows that were observed between 2013 and 2020 in the framework of a research study [14]. In line with the resistance data, penicillins, tetracyclines, sulfonamides and trimethoprim were the main substance classes used in sows during that period. For most of the other analysed substances, resistance was low and therefore a massive decrease in resistance could not be expected. It has to considered, that resistance data are only available for two non-consecutive years, separated by an eight-year gap. Hence, specific effects of the individual years may have contributed to the observed differences or to their absence. Further data from future years will be needed to fully understand the development.

The significant decrease in resistance to tetracycline and sulfamethoxazole is in line with the reduced sales of these substances [40] and their reduced use in sows [14]. However, a significant reduction of the percentage of resistance of *E. coli* was not observed for ampicillin and trimethoprim. The high relative share of penicillins and trimethoprim among all antibiotic treatments in sows may explain the non-decrease of resistance to ampicillin and trimethoprim [14] but do not explain the difference in the development in comparison to tetracycline and sulfamethoxazole. Further investigations are needed to analyse this discrepancy.

In contrast to *E. coli*, resistance in isolates of *Salmonella enterica* and in MRSA showed a heterogenous development. In *Salmonella enterica*, resistance to (fluoro)quinolones increased while no change in (fluoro)quinolone resistance was observed for *E. coli* and MRSA. It should be noted that only few *Salmonella* isolates could be tested, and since AMR in *Salmonella enterica* differs between serovars, a valid comparison of resistance rates is not possible. *Salmonella* serovar Derby clearly dominated the scene in sows in both years with 25.9% and 53.8% of the isolates in 2015 and 2023. This confirms previous data on *Salmonella* in breeding pigs in Germany and contrasts with the results from fattening pigs at slaughter that tend to have more *S*. Typhimurium, especially its monophasic variant [16].

### Changes in prevalence and AMR of MRSA and CTX^R^-*E. coli*

The prevalence of MRSA in the herds did not decrease between 2015 and 2023. In MRSA isolates, there was a non-significant (*p* > 0.05 but < 0.1) decrease in resistance to some substances (gentamicin, kanamycin and sulfamethoxazole). A significant decrease was only reported for resistance to chloramphenicol. On the other hand, there was a significant increase in resistance to other substances (trimethoprim, quinupristin/dalfopristin and tiamulin). The increase observed for trimethoprim was in line with the non-decrease observed in *E. coli*. As with *E. coli*, there is no obvious explanation for that as neither the use of trimethoprim nor that of tiamulin in sows did change substantially over time [14, 39]. A likely explanation is that resistance to tiamulin is linked to some other feature and therefore partially independent of the use. Looking at the association of the resistance traits of MRSA, we found that resistance to quinupristin/dalfopristin and to tiamulin were very closely linked, which is in line with the previous studies finding genes in staphylococci that code for resistance to both substance classes [46]. Likewise, there was a positive association between resistance to trimethoprim and to the two substances. Most isolates that were susceptible to tiamulin and/or quinupristin/dalfopristin were also susceptible to trimethoprim.

In a baseline survey in 2008, Germany was among the countries with the highest prevalence of MRSA in breeding pig herds with a prevalence of ≥ 40% of herds being positive [25]. The lower prevalence identified in our study may be associated with differences in the sampling methods. In the baseline survey, five dust samples were collected from different parts of the herd. In contrast, in our study, we only investigated one boot swab sample from the pregnant sow area. This approach was prone to lower sensitivity [47]. However, as we wished to exclude the potential effects of other age groups, i.e., suckling piglets and weaners, we could not follow the approach chosen in the baseline survey. In the baseline survey, most of the isolates were also from the *spa*-types t011 and t034 and few isolates were from *spa*-types that were not associated to CC398. However, the proportion of t034 was higher in 2015 and in 2023 as compared to the baseline survey and the proportion of t011 was lower. The association with the detection of MRSA in the farms with herd size is in line with the findings of the baseline survey in 2008 [24].

In contrast, CTX^R^-*E. coli* dropped in prevalence. There was no phenotypic resistance data in 2023 as the characterization of these bacteria had progressed to WGS based on CID (EU) 2020/1729. As most CTX^R^-*E. coli* in 2015 were MDR (84.8%), their selective advantage would be reduced by a broad reduction in antimicrobial consumption, which may have contributed to their reduced herd prevalence. In addition, a reduced use of 3rd and 4th generation cephalosporins was observed after 2018. Since 2018, German legislation requires susceptibility testing before these substances are used. As a consequence, their overall use in animals dropped significantly. This may have contributed to the reduction of the prevalence of CTX-R *E. coli* in the herds. According to a study on the period between 2013 and 2020 a substantial drop in the use of these substances was also observed in sows starting from 2018 [14]. However, 3rd and 4th generation cephalosporins were not used often in 2013 already. Interestingly, only one factor could be identified that was associated with AMR to individual substances in CTX^R^ *E. coli*: Isolates from farms that harboured MRSA were more likely to be resistant to ciprofloxacin. This is in line with the general association of MRSA and CTX^R^ *E. coli* detection in the herds.

The cephalosporin-resistance genes observed in breeding pigs in 2023 corresponded to those found in CTX^R^-*E. coli* from fattening pigs at slaughter in the same year [48]. There was a clear dominance of *bla*_CTX−M−1_ and a high frequency of *bla*_TEM−1_ in both, breeding herds and fattening pigs at slaughter, underlining the potential role of sows as reservoirs of *E. coli* harbouring these genes for the pork production chain.

### Changes in prevalence and AMR of *Salmonella*

Prevalence of *Salmonella* spp. was remarkably constant over time. In comparison with the baseline survey carried out in 2008, prevalence tended to be lower, even if compared to the pen prevalence in pregnant sows determined in that survey [49]. The overall prevalence cannot be validly compared as the sampling scheme was different and more intensive in 2008 than in 2015 and 2023. However, if only pen based sampling is considered, the data from 2008 and the current study are comparable: The prevalence found in the current study was lower than the pen prevalence observed in 2008 (6.4%). The baseline study also found no difference in *Salmonella* prevalence between production stages in the sow herd [50]. In Germany, there is a control program in place that addresses fattening pig herds, but sow herds are not directly covered [51]. This is in line with the assumption that control of *Salmonella* with respect to consumer protection is most effective when targeted at fattening pigs at slaughter or by interventions at slaughter [18]. Still, the authors mention that in high prevalence countries, also measures at the breeding herd level may be beneficial. In our study, *S*. Derby was the predominant serovar in both years which contrasts to the dominance of *S*. Typhimurium and its monophasic variant in fattening pigs at slaughter and pork at retail. The predominance of *S.* Derby is in line with the results of the European baseline survey in 2008, when *S.* Derby was also the most prevalent serovar in breeding pigs [49]. The difference between breeding and fattening pigs has been observed in other European countries as well [52]. This indicates that the sows are presumably not the main source of the *Salmonella* found in fattening pigs. There is rather circulation of *S.* Typhimurium within the weaner and fattening compartments [19, 53].

### Limitations

The two sampling frames in 2015 and 2023 were independent from each other, i.e. no direct comparisons between the individual herds sampled in 2015 and 2023 were possible. This was due to the nature of the monitoring that is not set up to follow individual herds but to reflect the national situation in the respective year. Another consequence of the use of data collected within a monitoring scheme is that only a limited amount of data on the farms can be collected. For example, farm specific data on AMU could not be collected. These would have been interesting when evaluating AMR. Finally, many people collecting the samples and many different laboratories are involved in such a study, which may be an additional source of variation that cannot be validly included in the analysis.

## Conclusions

Although breeding pigs were not in the focus of reduction programs for AMU in Germany, antimicrobial resistance in *E. coli* and the prevalence of CTX^R^-*E. coli* decreased in breeding herds between 2015 and 2023 in Germany. In contrast, there were no changes in the prevalence of *Salmonella* and MRSA. Resistance of *E. coli* to some antimicrobials was associated to the presence of MRSA in the herd (ampicillin, trimethoprim, tetracycline and multidrug resistance) and higher in larger herds (tetracycline, trimethoprim and sulfamethoxazole). Larger herds were also more prone to harbour MRSA and CTX^R^-*E. coli*, which may reflect the more intensive trade relations of larger herds with other herds increasing the risk of buying animals that carry resistant bacteria.

## Data Availability

The datasets used and/or analysed during the current study are available from the corresponding author on reasonable request.
